# The combined effects of konjac glucomannan and ultrasound treatments on the interaction of gluten and surimi protein

**DOI:** 10.1016/j.fochx.2026.103492

**Published:** 2026-01-12

**Authors:** Geng Cao, Zuoqian Yang, Xiangzheng Li, Xiaoyang He, Shuang Song, Chengrong Wen

**Affiliations:** a National Engineering Research Center for Seafood, State Key Laboratory of Marine Food Processing and Safety Control, Collaborative Innovation Center of Seafood Deep Processing, National Engineering Research Center of Seafood, National & Local Joint Engineering Laboratory for Marine Bioactive Polysaccharide Development and Application, School of Food Science and Technology, Dalian Polytechnic University, Dalian 116034, China; b School of Information Science and Engineering, Dalian Polytechnic University, Dalian 116034, China

**Keywords:** Gluten, Surimi protein, Ultrasound, Konjac glucomannan

## Abstract

The novel alternative of nutrition balance with plant and animal proteins is becoming the main component of nutrition in the modern diet. The properties of gluten, surimi and gluten/surimi mixture (GS) were analyzed after treatment with Konjac glucomannan (KGM), ultrasound (U) and Konjac glucomannan ultrasound (UKGM). Molecular weight, fluorescence intensity, intermolecular force, free sulfhydryl, microstructure, SDS-soluble protein and free amino acid content were investigated. All treatments increased the maximum fluorescence intensity (I_max_), free sulfhydryl (SH), and free amino groups of gluten. Treatment with KGM and ultrasound (U) decreased the SDS-soluble protein content, SH groups, and Imax of surimi protein, whereas the UKGM treatment showed the opposite trend. In the GS, all treatments decreased the I_max_ and SH groups, with UKGM reducing the SH content from 1.95 to 1.12 μmol/g. Consequently, the UKGM improved the interaction of gluten and surimi protein and resulted in better structural properties of the GS.

## Highlights

• Surimi protein, gluten, and their mixture were treated with konjac glucomannan, ultrasound, or a combination of both.
• Konjac glucomannan increased hydrogen bonds and ionic interactions of gluten.
• Ultrasound treatment decreased intrinsic fluorescence intensity and SDS-soluble protein of surimi protein.
• UKGM decreased SDS-soluble protein and free sulfhydryl content of gluten/surimi protein mixture.
• All treatments increased the contents SDS-soluble protein, free sulfhydryl and free amino group of gluten.

## 1 Introduction

Fish surimi protein, one of the sources of high-quality animal protein, is abundant in essential amino acids. Wheat gluten is a very important plant protein which easily aggregates and forms an integrated network skeleton structure after complete hydration (Qu et al., 2024). Nevertheless, wheat gluten is known to be deficient in lysine, which is one of the essential amino acids. In the context of food technology, proteins stand among the most important components of food, and the physicochemical properties and behavior of proteins during processing determine the quality of food (Alves & Tavares, 2019). According to protein sources, which are categorized into plant and animal proteins, animal raw materials such as milk, eggs, meat, and seafood remain the most important protein sources used by the food industry in the recent era, followed by plant sources such as cereal, oilseed, and legume proteins (Alves & Tavares, 2019; Lin & Miao, 2021).

The new food trend is the innovative alternative of plant and animal proteins with each other in food's main components as a result of ongoing changes in consumer preferences for traditional food. Gluten/surimi mixture (GS) products of surimi noodles based on surimi and wheat flour have attracted much attention (Cao et al., 2023). However, the alternative of different sources of proteins in the food system results in diverse effects, either synergistic or detrimental. *Alaskan pollock* surimi combined with soy protein and wheat gluten at specific ratios (e.g., 20–30% wheat gluten) has been reported to form structurally stable meat analogues under high-moisture extrusion conditions (Hou et al., 2023). Adding hydrolyzed wheat gluten could significantly improve the textural properties and thermal stability of surimi gels at high temperatures (Zhang et al., 2015). The improvement of destructive interactions and synergism between proteins in these systems is dependent on the physicochemical and its combined treatment. Therefore, much research has been done to improve the quality of a certain product by using additives such as polysaccharides (Cao et al., 2022), enzymes (Zhao et al., 2023), proteins (Hou et al., 2023), as well as physical methods such as ultrasound (Cao et al., 2023), electrical pulse (Beňo et al., 2023) and microwave (Wang et al., 2023). Additionally, several reported approaches integrate functional additives with physical techniques to achieve synergistic improvements in product quality.

Konjac glucomannan (KGM) is a type of food hydrocolloid and soluble dietary fiber composed of glucose and mannose in a molar ratio of 1:1.5–1:1.6 and polymerized by a β-1,4 glycosidic bond (Devaraj et al., 2019; Jian et al., 2016). KGM has been widely utilized in various foods such as wheat flour (Zhou, Cao, et al., 2013), meat (Gao et al., 2023), and their composites (Cao et al., 2022). The addition of 4.5% KGM disrupted the structure and strength of the continuous gluten network during dough formation (Guo et al., 2021). KGM was also found to improve the quality of surimi products and low-protein wheat flour (Cao et al., 2022; Zhou, Ma, et al., 2013). Our previous study demonstrated that high-viscosity KGM significantly improved the structural and textural properties of wheat–surimi noodles, as evidenced by enhanced starch–gluten–surimi network integrity, and improved noodle hardness, tensile length, and cooking quality (Cao et al., 2022).

Ultrasound treatment is an environmentally friendly and non-thermal physical technique and is considered a superior alternative to conventional thermal processing methods in food applications. It is widely used in the food industry due to its thermal, mechanical, and chemical effects (Mirmoghtadaie et al., 2016). In particular, it is used to modify proteins and protein complexes. Multiscale ultrasound strengthened the binding and electrostatic interaction between whey protein and pullulan (Qayum et al., 2024). Ultrasound in combination with linseed gum improved the foaming of egg gel and increased the surface tension (Tian et al., 2024). Ultrasound pretreatment combined with KGM modification increased the content of free sulfhydryl groups and disulfide bonds, and improved the freezing tolerance of dough (Lu et al., 2023), and promoted the unfolding and moderate thermal aggregation of low-salt myofibrillar protein and gel strength of the compound gel (Gao et al., 2023). However, the mechanism of meat and plant gel enhancement by ultrasound combined with polysaccharides is still poorly understood.

In previous studies (Cao et al., 2022; Cao et al., 2023), we improved the texture of conventional surimi-wheat noodles by KGM and ultrasound-assisted resting. What's more, it is really necessary to understand the mechanism for the effects of KGM and ultrasound on the surimi-wheat noodles at the microscopic level. Therefore, this study focused on the changes in the protein structure of gluten, surimi, and GS by konjac glucomannan, ultrasound, and konjac glucomannan-ultrasound treatment. In addition, the mechanism of protein changes in terms of structural properties, covalent and non-covalent interaction, protein conformation, and molecular interaction was elucidated. This research would provide a valuable reference for the novel alternative of plant and animal proteins in the main ingredients of food and fulfill the processing needs of the food industry.

## 2 Materials and methods

### 2.1 Materials

High-gluten wheat flour was provided by COFCO International (Beijing) Co., Ltd. (Beijing, China). Konjac powder (food grade) with a viscosity of approximately 32,000 mPa·s was obtained from Zhejiang Yinuo Biotechnology Co., Ltd. (Lanxi, China). Frozen long-finned herring surimi (water content: 74.5%, protein content: 9.06%, food grade) was sourced from Hubei Honghu Jingli Aquatic Food Co., Ltd. (Honghu, China). Salt was from China National Salt Industry Group Co., Ltd. (Beijing, China). Additionally, fluorescein isothiocyanate (FITC, analysis level), trichloroacetic acid (TCA, analysis level), and β-mercaptoethanol (analysis level) were obtained from Aladdin Biochemical Technology Co., Ltd. (Shanghai, China). Ethylene Diamine Tetraacetic Acid (EDTA, analysis level) and Rhodamine B (analysis level) were obtained from Macklin Biochemical Co., Ltd. (Shanghai, China). Trimethylol aminomethane (Tris, analysis level) was obtained from Solarbio Science & Technology Co., Ltd. (Beijing, China). This study was conducted between March and August 2022 at Dalian Polytechnic University, China.

### 2.2 Preparation of gluten and surimi protein

Wheat gluten was prepared according to the method reported by Guo et al. (2021). Wheat flour was defatted by dispersing in chloroform (*w*/*v*, 1:2) and continuous stirring for 20 min at 25 °C. The suspension was then filtered and defatted three times using a Buchner funnel and dried in a fuming cupboard. The defatted flour and salt solution (0.4 M, 200 mL) were mixed in a kneading machine (HM740, Hanshang Co., Ltd., Qingdao, China) and kneaded into dough at medium speed for 10 min. After resting at 37 °C and 80% relative humidity for 10 min, the dough was washed with NaCl solution until crude wet gluten was obtained. The gluten was then washed with deionized water, freeze-dried (Scientz-30ND, NingBo Scientz Biotechnology Co., Ltd., Ningbo, China), and ground through a 200-mesh sieve for further analysis.

Surimi proteins were extracted according to the method described by Ma et al. (2019). Surimi was thawed at 4 °C for 12 h, mixed with 10-fold deionized water, and homogenized for 30 min (JYL-Y5, Joyoung Co., Ltd., Jinan, China). The resulting slurry was conducted by adjusting the pH of the sample to 10.5 (2 M NaOH). After continuous stirring for 2 h at 4 °C, the mixture was centrifuged (8000 *g*, 30 min, and 4 °C). The pH of the supernatant was adjusted to 5.5 (2 M HCl) to precipitate the proteins. The obtained supernatant was adjusted to pH 4.5 and centrifuged again (10,000 *g*, 15 min, and 4 °C). The protein precipitate was suspended in deionized water (pH 7.0). The protein suspension was stirred overnight at 4 °C and then freeze-dried.

About 1 g of surimi protein, gluten, and surimi protein/gluten blends in a ratio of 1:1 (GS) was mixed with or without KGM (*w*/w, 3%) in 5 mL water, respectively. The mixture was kneaded to a uniform mass, placed in a centrifuge tube, and then fully hydrated at 4 °C for 1 h. Then the sample was put in an ultrasound water bath (KQ-500DE, Kunshan Ultrasonic Instrument Co., Ltd., Kunshan, China) treated at 21.33 W/L at 35 °C for 30 min. After that, sample was immediately frozen in liquid nitrogen and freeze-dried for later analysis. Samples consisting of untreated wheat gluten, surimi protein, and their gluten-surimi (GS) mixture without KGM addition or physical treatment were abbreviated as the control. Other treatments were denoted as KGM (protein–KGM mixtures), U (proteins treated by ultrasound), and UKGM (protein–KGM mixtures treated by ultrasound).

### 2.3 Measurement of protein content

To prepare the samples, 75 mg of freeze-dried powder and 1 mL of buffer solution (1% SDS, phosphate solution, pH 7.4) were mixed and stirred completely at 25 °C for 1 h. The mixtures were centrifuged at 10,000 *g* for 10 min at 4 °C obtain the supernatant. The protein content was measured using the BCA protein assay kit (Beijing Solarbio Science & Technology Co., Ltd., Beijing, China).

### 2.4 Determination of the intrinsic fluorescence spectra

The value of the intrinsic fluorescence spectrum was determined by modifying the method of Si et al. (2021). The intrinsic fluorescence spectra of 0.1% (*w*/*v*) sample solution (phosphate buffer, 0.05 M, pH 7.0) was measured using a fluorescence spectrophotometer (F-2700, Hitachi, Ltd., Tokyo, Japan) under excitation wavelength 280 nm, recorded spectral range 300–400 nm, and excitation slit 5 nm at 25 °C.

### 2.5 Sodium dodecyl sulfate-polyacrylamide gel electrophoresis (SDS-PAGE)

A SDS-PAGE method was performed using a 12.5% PAGE Gel Fast Preparation Kit (Shanghai Epizyme Biomedical Technology Co., Ltd., Shanghai, China). Samples (5 mg) were stirred in 1 mL of loading buffer and then were heated until the samples completely dissolved. The supernatant (10 μL) and mark were loaded into each lane (Guo et al., 2021). Runs were performed first at 80 V for 10 min and then at 120 V for 60 min. The gel was stained in Coomassie brilliant blue G-250 (solution analysis level) for 5 h and then decolorized with decolorization solution (250 mL of 95% ethanol and 80 mL of glacial acetic acid, analysis level) until the protein was clear. A densitometer (MF-ChemiBIS 2.0, DNR Bio-Imaging Systems Ltd., Jerusalem, Israel) was used to take images of the gels.

### 2.6 Free amino group (NH_2_) determination

Determination of NH_2_ content was carried out after modifying the reported method (Du et al., 2020). The o-phthaldialdehyde (OPA) solution was prepared with 80 mg OPA, 3.81 g di‑sodium-tetraborate decahydrate, and 100 mg sodium dodecylsulfate per 100 mL. 40 mg of sample was dispersed in 3 mL of HCl solution (0.1 mol/L, pH 1.0) and stirred for 30 min, and then centrifuged at 10,000 *g* for 10 min at 4 °C. 20 μL of supernatant was mixed with 150 μL of OPA and 2-mercaptoethanol (*v*/v = 21.27:1). The absorbance at 340 nm was recorded using a microplate reader, and a leucine standard curve was used.

### 2.7 Measurement of protein solubility

Determination of the protein content in different selective solutions to cleave the specific interactions was carried out after some modification of the method of Si et al. (2021). Based on 0.05 M phosphate buffer (pH 7.0), the following solutions were used: 0.1 M NaCl (S1), 0.6 M NaCl (S2), 0.6 M NaCl +1.5 M urea (S3), and 0.6 M NaCl +8 M urea (S4). The samples (200 mg) and each reagent (S1-S4) were mixed and stirred for 1 h at 25 °C. The mixtures were centrifuged at 10,000 *g*, 10 min, 4 °C. The protein concentration of the supernatant was determined by a BCA assay kit. The contributions of ionic-bond, hydrogen-bond, and hydrophobic interactions to dough proteins were assessed by comparing protein concentrations between successive extracts (S1, S2, S3 and S4).

### 2.8 Measurement of free sulfhydryl (SH) content

The free sulfhydryl (SH) content was measured according to Cao et al. (2023). Sample preparation: Approximately 75 mg of freeze-dried samples and buffer solution were stirred with 4 mL buffer solution (86 mmol/L Tris-Glycine buffer solution, 90 mmol/L Glycine, 4 mmol/L EDTA, and 4.7 g of guanidine hydrochloride) for 30 min. Free sulfhydryl content: The mixtures were centrifuged (13,600 *g*, 4 °C, 15 min) to get the supernatant, then 40 μL Ellman's reagent (4 mg/mL DTNB) and 5 mL of urea solution (8 mol/L urea, Tris-Glycine buffer) were added. All samples were incubated for 30 min. The absorbance at 412 nm was measured with the same concentration of Ellman's reagent as blank. The equations for free sulfhydryl groups are shown in Eq.1 respectively.

SH (μmol/g) =$\frac{73.53\times A_{412}\times 5.02}{c.}$

Where SH is the free sulfhydryl content, A_412_ is the absorbance of the sample at 412 nm, C is the sample concentration (15 g/L).

### 2.9 Scanning electron microscopy

Variations in the protein structure and degeneration of samples were observed using a JSM-7800F SEM (JSM - 7800F, JEOL Ltd., Tokyo, Japan). Samples were freeze-dried, broken up, and then coated with gold before being inspected at a 5.0 kV accelerating voltage and magnifications of up to 500 × .

### 2.10 Statistical analysis

The values were shown as means ± standard after each measurement was made independently-thrice. One-way ANOVA and Tukey's test were adopted to measure significant differences by using IBM SPSS Statistics 24 (SPSS Inc., Chicago, USA). The average value was deemed significant when *P < 0.05* in a statistical analysis.

## 3 Results and discussion

### 3.1 Sodium dodecyl sulfate (SDS) protein

**Fig. 1:**
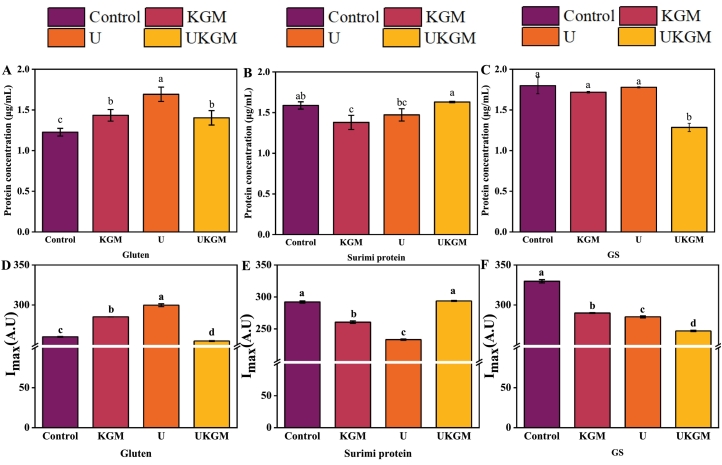
The soluble protein content and intrinsic fluorescence intensity (I_max_) of gluten, surimi protein, and GS treated with KGM, U and UKGM. Different letters superscripted on the columns indicate significant differences (*P < 0.05*).

The sodium dodecyl sulfate (SDS) molecule causes protein denaturation by altering the original maintenance of the protein's three-dimensional and quaternary structure (Andersen et al., 2009; Sonesson et al., 2007), leading to changes in protein solubility and protein aggregation. U, KGM, and UKGM treatments lead to a significant (*P* < 0.05) increase in gluten solubility (Fig. 1A), with the highest value for U treatment, but there was no significant difference between KGM and UKGM. It implies that all three treatments were capable of preventing the aggregation of gluten fractions. Mironeasa et al. (2019) reported that ultrasound and KGM can disrupt the network structure of gluten aggregate and integration after full hydration. Research also found that the sonication technique could break down large particles at the mesoscopic level and favor a homogenous dispersion of proteins without causing polymer degradation and disrupt major non-covalent and covalent interactions of gluten (Marcuzzo et al., 2010). The solubility of surimi protein in the treatment groups (KGM, U, and UKGM) varied (Fig. 1B), with only a significant (*P* < 0.05) decrease in KGM treatment. It is probably because KGM fills and entangles the structure of the surimi protein (Iglesias-Otero et al., 2010), which does not have a continuous structure like gluten proteins (Qu et al., 2024), and forms aggregation to decrease the solubility, but ultrasound prevented the interaction (Lu et al., 2023). There was no noticeable difference in the solubility of GS with KGM and U treatment and a significant (*P* < 0.05) decrease in UKGM treatment (Fig. 1C). The GS seemed to show high tolerance to UKGM treatment compared to U and KGM treatments.

### 3.2 Intrinsic fluorescence spectra analysis

The intrinsic fluorescence of proteins offers information regarding the microenvironment of fluorescent amino acids, such as tyrosine and tryptophan residues, serving as a notable indicator to characterize the conformation, kinetics, and intermolecular interactions of proteins (Si et al., 2021; Zhou, Cao, et al., 2013). Fig. 1D, E, and F illustrate changes in the maximum fluorescence intensity (I_max_) of gluten, surimi, and GS after KGM, U, and UKGM treatments, with shifts reflecting structural alterations in the proteins. For gluten, I_max_ increased significantly (*P < 0.05*) with KGM and U treatments but decreased significantly (*P < 0.05*) under UKGM treatment. The U treatment resulted in the highest I_max_, indicating the strongest exposure of tryptophan and tyrosine residues due to modifications in the tertiary structure of gluten molecules and a loosening of the structure in the shell (Liu et al., 2022). Interestingly, UKGM unexpectedly reduced the maximum fluorescence intensity, possibly due to ultrasound-induced conformational changes in KGM, which promote its entanglement with protein molecules, shielding fluorescent groups and limiting their mobility (Gao et al., 2023). Previous research has demonstrated that ultrasound treatment induces changes in protein structure due to modifications in the spatial arrangement and interactions of amino acids (Zhang et al., 2021). Furthermore, KGM might infiltrate the hydrophobic region of gluten, increasing the contact between tryptophan residues, and resulting in an increase in the polarity of the microenvironment of the residues (Iglesias-Otero et al., 2010). Compared to the control, when KGM was added to surimi protein or ultrasound treatment was applied, the I_max_ value decreased significantly (*P* < 0.05), while the UKGM treatment had no obvious effect (Fig. 1E). The distinct effects of the treatments on gluten and surimi protein can be attributed to the different compositions of gluten and surimi protein. All treatments (U, KGM, and UKGM) witnessed a decrease in I_max_ in GS (Fig. 1F), which suggested that the unfolded protein molecules were more prone to aggregate within themselves or with KGM. This phenomenon can be attributed to the loose structure of GS and the reduction of the microenvironment polarity of aromatic side chains like tryptophan and tyrosine residues caused by KGM, U, and UKGM treatments (Si et al., 2021).

### 3.3 Free sulfhydryl content analysis

Sulfhydryl groups represent a key functional moiety in both gluten and myofibrillar proteins, with free SH groups involved in thiol-exchange reactions (Mi et al., 2024). Table 1 illustrated that surimi protein had the highest free SH content, followed by GS and gluten., leading to the formation of covalent bonds between the two molecules (Nand et al., 2018). On behalf of different treatments, UKGM, KGM, and U treatments led to a significant increase (*P < 0.05*) in sequence in the content of free thiols in gluten. An increase in free SH groups in gluten, reflecting a weakening of the covalent cross-linking between gluten molecules (Yang et al., 2023). This is possibly because KGM, U, and UKGM treatments disrupted the continuous structure of gluten proteins and inhibited the formation of the covalent cross-linking. The content of free SH in surimi protein declined significantly (*P < 0.05*) from 3.42 μmol/g to 2.21 and 2.92 μmol/g with KGM and U treatments. In contrast, the UKGM treatment had an opposite effect. KGM filled the gap junction between surimi proteins, and ultrasound altered the spatial structure of proteins, leading to protein aggregation. When KGM and ultrasound treatment were combined, KGM interacted with surimi protein molecular chains, which were unfolded by ultrasound, thereby the content of inter- and intra-molecular SH content rose. The free SH contents in GS decreased significantly (*P < 0.05*) under KGM, U, and UKGM treatment, suggesting that KGM and ultrasound treatment could enhance the interaction between and within gluten and surimi protein. This might be attributed to the rearrangement of intermolecular forces between gluten molecules, promoting the covalent cross-linking of proteins (Yang et al., 2023).

**Table 1:** The free SH content of gluten, surimi protein, and GS treated with KGM, U and UKGM.

	Gluten (μmol/g)	Surimi protein (μmol/g)	GS(μmol/g)
Control	0.22 ± 0.01^d^	3.42 ± 0.01^b^	1.95 ± 0.01^a^
KGM	0.36 ± 0.01^b^	2.21 ± 0.01^d^	1.29 ± 0.01^b^
U	0.43 ± 0.02^a^	2.92 ± 0.01^c^	1.18 ± 0.01^c^
UKGM	0.31 ± 0.01^c^	3.60 ± 0.01^a^	1.12 ± 0.01^d^

Different letters superscripted on the columns indicate significant differences (*P < 0.05*).

### 3.4 Free amino acid content analysis

**Fig. 2:**
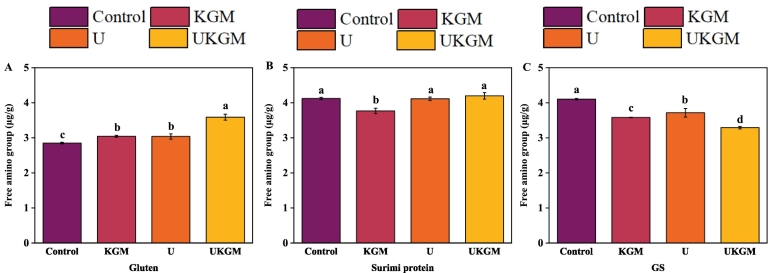
The free amino content of gluten, surimi protein, and GS treated with KGM, U and UKGM. Different letters superscripted on the columns indicate significant differences (*P < 0.05*).

The reaction between the –NH_2_ group in the side chain of the amino acid and the carboxyl group in gluten and surimi protein also plays an important role in the blend system. According to the results presented in Fig. 2, U, KGM, and UKGM treatments significantly increased (*P < 0.05*) the amounts of free amino acids found in gluten while decreasing in GS. On the other hand, there was no significant change in the content of free amino acids in surimi protein, except for a significant decrease (*P < 0.05*) when KGM was added. KGM inhibited the interaction of gluten while enhancing the interaction of surimi protein and surimi protein with gluten. When exposed to ultrasound, protein unfolding and acoustic cavitation can cause the structural and conformational changes of gluten and surimi protein, which enhanced the interaction between gluten and surimi protein in GS, leading to the decrease of the –NH_2_ group (Lu et al., 2023). This phenomenon was consistent with the above results of the changes in the fluorescence intensity of the samples.

### 3.5 Chemical interactions analysis

**Fig. 3:**
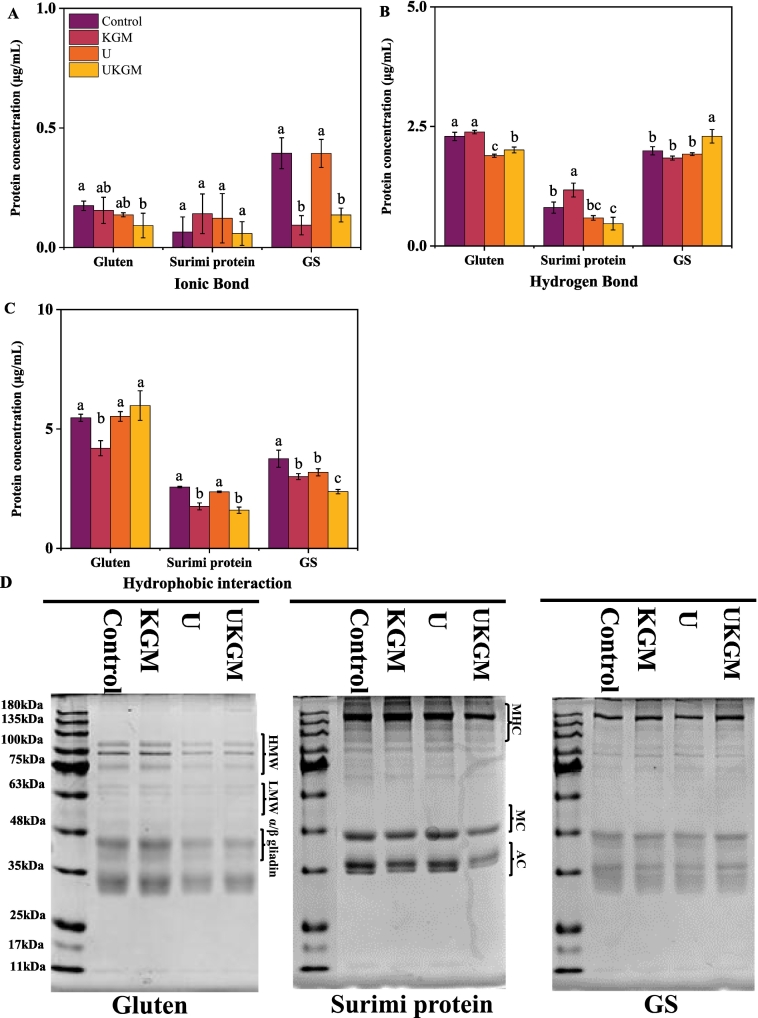
The concentration and SDS-PAGE electrophoretograms of gluten, surimi protein, and GS treated with KGM, U and UKGM. (A: ionic bond, B: hydrogen bond C: hydrophobic interaction. HMW: high molecular weight gluten subunit; LMW: low molecular weight gluten subunit; α/β-gliadin; MHC: myosin heavy chain; MC: myosin chain; AC: Actin). Different letters superscripted on the columns indicate significant differences (*P < 0.05*).

Peptide bonds, hydrogen bonds, and hydrophobic interactions are vital intermolecular forces that assist in maintaining the complex three-dimensional structure of proteins. The lower concentration in Fig. 3A (S1: 0.05 M NaCl and S2: 0.6 M NaCl) indicated fewer ionizable amino acid residues and weaker ionic interactions within the protein (Si et al., 2021). According to Fig. 3A, the solubilities of gluten, surimi, and GS were significantly (*P < 0.05*) changed when treated by KGM, U, and UKGM. All treatments of gluten proteins exhibited a decreasing trend in ionic bonds, indicating a reduction in ionizable amino acid residues and suggesting that ionic interactions were the weakest among the tested forces. For surimi proteins, no significant differences were observed; however, KGM and U treatments showed an increasing trend compared to control and UKGM. When GS was treated by KGM and UKGM, their ionic bond significantly (*P < 0.05*) decreased compared to control and KGM. This is likely due to the increased spatial hindrance caused by KGM, which weakens the ionic interactions of proteins, resulting in weak interactions of ionizable amino acid residues. This effect is similar to that observed by (Si et al., 2021), who used arabinoxylan in gluten protein. The concentration of gluten and GS in Fig. 3A after U treatment was lower than that in the control group, possibly due to the hindering effect of KGM on the solubility of ionizable amino acid residues. The solubility of GS was significantly (*P < 0.05*) decreased with UKGM and KGM treatment, whereas the concentration of GS was significantly (*P < 0.05*) increased when compared to the control. Surimi protein was unable to form a dense and continuous protein structure, and the -OH in KGM interacted with the side chains of amino acids. Meanwhile, ultrasound increase the number of carboxyl and amino groups of acidic and basic amino acid branches by modifying its spatial structure and expanding the previously discontinuous structure (Liu et al., 2022).

The protein concentration differences in Fig. 3B and Fig. 3C (S2 (0.6 M NaCl), S3 (0.6 M NaCl +1.5 M urea, S4 (0.6 M NaCl +8 M urea)) reflect the contribution of hydrogen bonds and hydrophobic interactions, as urea disrupts these forces and thereby promotes protein solvation (Si et al., 2021). The protein concentration of gluten, surimi protein, and GS in Fig. 3B and Fig. 3C were higher than Fig. 3A, which indicated that hydrogen bonds and hydrophobic interactions were the primary molecular forces in these protein systems. The addition of KGM increased the concentration of surimi protein in Fig. 3B by acting as a spatial filler within the relatively discontinuous protein environment, thereby serving as a scaffold that enhances hydrogen bonding between protein molecules. However, the solubility of gluten and surimi proteins in Fig. 3B decreased after U treatment due to alterations in hydrogen bonding, which may result from the disruption of intramolecular chemical bonds, loosening of peptide chains, and enhanced hydrophobic interaction. Compared to control, no significant changes were observed in GS treated by KGM and ultrasound, but the hydrogen bond concentration of GS with UKGM in Fig. 3B was significantly higher than that of Control, KGM and U treatment. UKGM treatment significantly (*P < 0.05*) increased the solubility of GS. On the one hand, this was achieved by enhancing water dispersibility in the microenvironment through the competition between KGM and water molecules, which strengthened the binding of KGM to water via -OH and H atoms, thereby increasing hydrogen bonding interactions between proteins. On the other hand, ultrasound (U) treatment may change the protein state, causing them to become entangled or isolated, which in turn exposes the important core regions of the proteins. Overall, UKGM treatment induces changes in protein solubility through the synergistic effects of water molecule distribution and protein structural state. With the increase in urea concentration, all treatments reduced the hydrophobic interaction of GS in Fig. 3C, while only U and UKGM treatments could significantly (*P < 0.05*) decreasing the hydrophobic interaction of surimi protein. In Fig. 3C, treatment with KGM significantly (*P < 0.05*) reduced the protein concentration of gluten proteins, whereas ultrasound-treated KGM (UKGM) resulted in a notable increase in hydrophobic interaction. It acts not only as a space filler and exposes buried hydrophobic groups in the protein molecules, promoting the formation of new disulfide connections between free sulfhydryl groups, but also bonds to other gluten via Van der Waals forces (Lu et al., 2023). The mechanical force of ultrasound broke the Van der Waals forces holding protein molecules together (Liu et al., 2022). Additionally, the hydroxyl and acetyl groups introduced by KGM might change the interaction of hydrogen bonds between the surimi proteins.

### 3.6 Sodium dodecyl sulfate-polyacrylamide gel electrophoresis analysis

Sodium dodecyl sulfate-polyacrylamide gel electrophoresis (SDS-PAGE) under denaturing and reducing conditions were used to investigate the influences of U, KGM, and UKGM treatments on the structure of gluten, surimi protein, and GS. There were no obvious differences and new bands in the number and location of the reduced electrophoretic bands for gluten, surimi protein, and GS (Fig. 3E), indicating that U, KGM, and UKGM treatment did not change the subunits or formed new protein structure. The intensity of the gluten spectrum is similar to the results of Zhang et al. (2023), with no significant differences in the molecular size distribution in the gluten spectrum. Generally, the treatments did not cause major alterations in the gluten electrophoresis bands. In gluten proteins, a more intense band around 135 kDa was observed under UKGM treatment compared to other groups. This increase in band intensity may be attributed to enhanced protein aggregation or cross-linking between gluten subunits, potentially promoted by ultrasound-induced unfolding and subsequent interactions with KGM. However, a decrease in band intensity between 180 and 135 kDa was observed in surimi proteins following UKGM treatment, suggesting that this treatment disrupted protein aggregation. This observation is consistent with our previous intrinsic fluorescence spectroscopy results, which indicated conformational changes and reduced intermolecular interactions in surimi proteins after UKGM treatment (Gao et al. 2023). The intensity in the HMW region (180–135 kDa) of GS was slightly reduced with the addition of KGM and ultrasound treatment. However, a notable increase in band intensity was observed under UKGM treatment, indicating that the combination of KGM and ultrasound may synergistically promote the formation of high-molecular-weight protein complexes. Overall, UKGM treatment appeared to promote protein aggregation in GS, whereas it exerted an opposite effect on surimi protein by disrupting aggregation and reducing high-molecular-weight complexes (Cao, et al., 2013).

### 3.7 Microstructural analysis

**Fig. 4:**
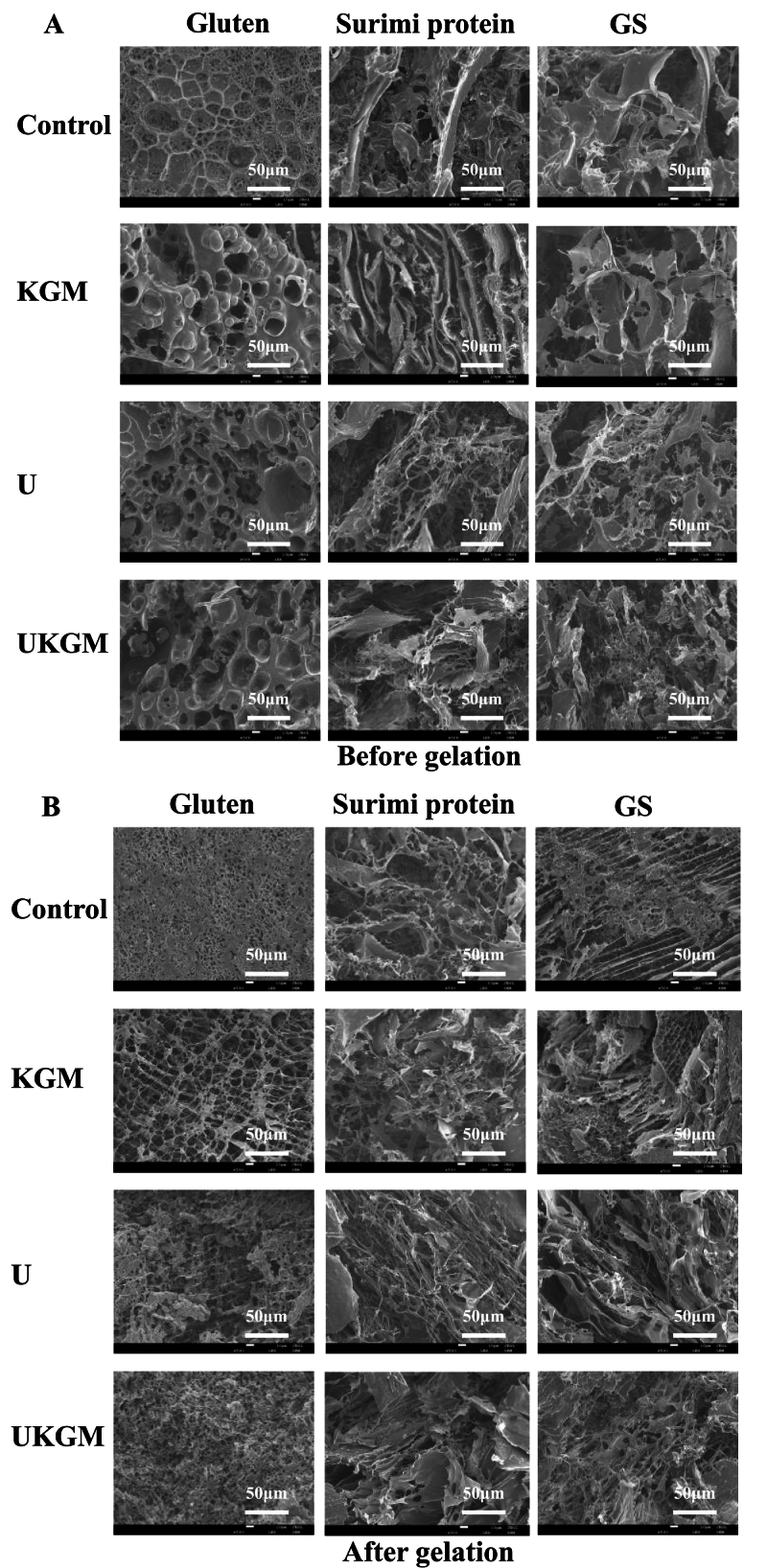
SEM images of gluten, surimi protein, and GS treated with KGM, U and UKGM before (A, × 500) and after (B, × 500) gelation.

The impact of KGM, U, and UKGM treatments on the microstructure of gluten, surimi, and GS before and after gelation was observed, as depicted in Fig. 4. All three treatments disrupted the gluten network, resulting in large, irregular, and coarse holes (Fig. 4A). After gelation, the control group exhibited a dense and continuous structure (Fig. 4B), whereas gluten treated with KGM and ultrasound displayed less dense networks. UKGM treatment produced smaller, denser holes, indicating a more compact structure. For the control surimi protein, large layered structures with porous structures at the bottom were observed. KGM thickened this structure, while ultrasound created a loose network with filamentous materials. The UKGM treatment led to a framework structure that was both layered and looser. Post-gelation, the surimi protein framework became thinner, with ultrasound treatment promoting a filamentous structure and UKGM resulting in a denser arrangement. When GS was treated with and without UKMG, gluten and surimi proteins partially detachment before heating. However, the addition of KGM and ultrasound treatment created large gaps within the GS structure. Upon heating, the GS structure contained both large and small holes, while the GS with KGM exhibited a layered structure with some protein coverage. This suggests that KGM adhered to the protein surface, causing partial detachment (Zhou, et al. 2013, Guo, et al. 2023). Conversely, ultrasound and UKGM treatments disrupted the GS structure into random filamentous configurations. Ultrasound treatment contributed to the separation of protein structures, leading to the formation of filamentous gluten after gelatinization. Previous studies also indicate that UKGM may lead to a denser myofibril structure (Gao et al., 2023).

### 3.8 Mechanisms

**Fig. 5:**
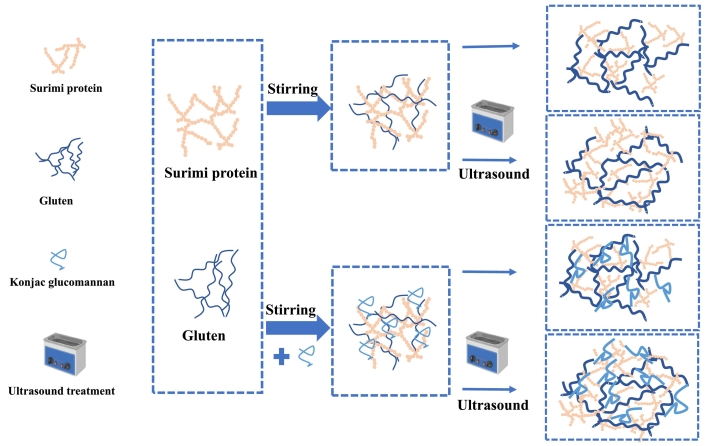
The mechanism of konjac glucomannan or/and ultrasound treatment on the interaction between surimi and gluten protein.

Based on the experimental observations obtained in this study, a possible interaction mechanism is proposed to explain how konjac glucomannan (KGM) and ultrasound treatment modulate the structure of the surimi–gluten protein system (Fig. 5). When hydrated and mixed, gluten proteins tend to self-aggregate and form a relatively continuous network, whereas surimi proteins exhibit a more heterogeneous and discontinuous structure, resulting in an uneven internal microenvironment with large voids (Li et al., 2020). Consequently, the blended surimi–gluten system displays poor structural uniformity.

The incorporation of KGM is suggested to contribute to network rearrangement by filling these voids, which can be attributed to its high water-holding capacity and abundant hydroxyl groups. By absorbing and redistributing water within the system, KGM may influence protein spatial organization and promote a more compact and homogeneous network structure, as reflected by the observed changes in water distribution and microstructural density (Guo et al., 2021; Zhao et al., 2023).

Ultrasound treatment is likely to induce partial unfolding of surimi–gluten proteins through cavitation effects, thereby facilitating the reorganization of intermolecular interactions. Previous studies have shown that ultrasound can disrupt non-covalent interactions while increasing molecular mobility, which may enhance protein–protein interactions and aggregation behavior (Gao et al., 2023; Marcuzzo et al., 2010). In the present system, these effects are supported by variations in rheological properties and free sulfhydryl content, indicating structural rearrangement at the molecular level.

When KGM and ultrasound are applied in combination, their synergistic effects may further promote the rearrangement of intermolecular forces, including non-covalent interactions and possible covalent crosslinking. The steric hindrance of KGM, together with ultrasound-enhanced molecular collisions, may increase the probability of interactions between sulfhydryl and amino groups, leading to decreased free SH and free amino group contents and the formation of new crosslinks (Lu et al., 2023). Collectively, these effects contribute to the formation of a denser and more stable surimi-gluten protein network, ultimately improving the microstructural integrity and macroscopic properties of the system.

## 4 Conclusion

A series of experiments on the protein properties and structure were conducted to evaluate the effects of ultrasound treatment combined with KGM on the interactions between gluten and surimi protein. The findings reveal that this combination enhances the structure of the surimi–gluten mixture. Additionally, ultrasound alters the microenvironment of protein molecules, facilitating access to hydrophobic areas and increasing fluorescence intensity, as observed in maximum fluorescence intensity assays. UKGM treatment reduced the free sulfhydryl content of the GS from 1.95 to 1.12 μmol/g indicating enhanced intermolecular interactions and protein rearrangement. Meanwhile, t free sulfhydryl content of surimi protein increased from 3.42 to 3.60 μmol/g after UKGM treatment. Changes in free sulfhydryl content further supported these structural modifications. The application of UKGM modified the conformation and aggregation behavior of GS through non-covalent interactions, while ultrasound treatment led to a looser protein structure in the absence of KGM. Overall, KGM played an essential role in reorganizing intermolecular interactions among GS during ultrasound treatment. These insights highlight the potential benefits of utilizing ultrasound and KGM to improve product properties in surimi-wheat blends.

## CRediT authorship contribution statement

**Geng Cao:** Writing – original draft, Methodology, Investigation. **Zuoqian Yang:** Investigation, Formal analysis. **Xiangzheng Li:** Investigation. **Xiaoyang He:** Methodology, Investigation. **Shuang Song:** Resources. **Chengrong Wen:** Writing – review & editing, Resources, Funding acquisition, Data curation, Conceptualization.

## Declaration of competing interest

The authors declare that they have no known competing financial interests or personal relationships that could have appeared to influence the work reported in this paper.

## Data Availability

The authors do not have permission to share data.
